# Does the engagement of clinicians and organisations in research improve healthcare performance: a three-stage review

**DOI:** 10.1136/bmjopen-2015-009415

**Published:** 2015-12-09

**Authors:** Annette Boaz, Stephen Hanney, Teresa Jones, Bryony Soper

**Affiliations:** 1 Faculty of Health, Social Care and Education, St George's, University of London and Kingston University, Grosvenor Wing, Cranmer Terrace, London, UK; 2 Health Economics Research Group, Brunel University London, London, UK

## Abstract

**Objective:**

There is a widely held assumption that engagement by clinicians and healthcare organisations in research improves healthcare performance at various levels, but little direct empirical evidence has previously been collated. The objective of this study was to address the question: Does research engagement (by clinicians and organisations) improve healthcare performance?

**Methods:**

An hourglass-shaped review was developed, consisting of three stages: (1) a planning and mapping stage; (2) a focused review concentrating on the core question of whether or not research engagement improves healthcare performance; and (3) a wider (but less systematic) review of papers identified during the two earlier stages, focusing on mechanisms.

**Results:**

Of the 33 papers included in the focused review, 28 identified improvements in health services performance. Seven out of these papers reported some improvement in health outcomes, with others reporting improved processes of care. The wider review demonstrated that mechanisms such as collaborative and action research can encourage some progress along the pathway from research engagement towards improved healthcare performance. Organisations that have deliberately integrated the research function into organisational structures demonstrate how research engagement can, among other factors, contribute to improved healthcare performance.

**Conclusions:**

Current evidence suggests that there is an association between the engagement of individuals and healthcare organisations in research and improvements in healthcare performance. The mechanisms through which research engagement might improve healthcare performance overlap and rarely act in isolation, and their effectiveness often depends on the context in which they operate.

**Strengths and limitations of this study**

- This review brings together for the first time a diverse body of literature addressing whether engaging clinicians and healthcare organisations in research is the likely to improve healthcare performance.
- It also explores the mechanisms through which improvement is achieved to try and understand how any improvements might come about.
- However, it relies on the quality and coverage of the existing literature.
- It is an extremely complex topic, but nonetheless one worthy of further exploration, particularly given the pressure to justify research spending in healthcare systems, and to encourage its implementation.

## Background

There is a widely held assumption that engagement by clinicians and healthcare organisations in research improves healthcare performance at various levels,^1^ ^2^ but little direct empirical evidence has previously been collated. A previous review (published in 2011) looked at the effects on patients of their healthcare practitioner’s or organisation’s participation in clinical trials.^3^ It identified 13 papers and suggested that the evidence to support a positive association was less strong than previously thought. Another paper, published in the same year, reported that participants at an international workshop held in 2009 had also concluded that the literature on the impact of research activity on the quality of healthcare outcomes within research-active institutions and healthcare systems in general was not extensive.^4^

This current paper reports on a literature review conducted to map and explore plausible mechanisms through which research engagement might improve healthcare performance at clinician or organisational level. The review addressed the question, “Does research engagement by clinicians and organisations improve healthcare performance?”, and also sought to identify the mechanisms that might be involved. Despite the obvious overlaps, this question is different from that of whether individual patients benefit from trial participation on which the 2009 workshop also commented, concluding that on this second issue there was, in contrast, a ‘substantial literature’ but still, nonetheless, a lack of conclusive evidence.^4^

### Theoretical context

Numerous theoretical perspectives have a bearing on our research question. For example, research engagement has been conceptualised as a way of increasing the ability and willingness of various groups of stakeholders to use research. This includes the theory of absorptive capacity, which seeks to explain how conducting research and development (R&D) within an organisation can help that organisation develop and maintain its broader capabilities to assimilate and exploit externally available information from research.^5^ There is also literature focusing on the characteristics of individual research adopters,^6^ ^7^ and work on clinical leadership and the role of medical academics.^8^

Other bodies of relevant theory seek to underpin efforts to explore how better co-ordination of research engagement might enhance the effectiveness of research, including the development of research networks^9^ ^10^ and initiatives designed to re-shape relations between clinical research and healthcare delivery systems such as the NIH Road Map in the USA,^11^ the NIHR Collaborations for Leadership in Applied Health Research and Care (CLAHRCs)^12^ and the Academic Health Science Networks^13^ in the UK, and similar developments in other countries.^14^ ^15^ Research engagement has also been conceptualised as a way of ensuring that research is used to improve the healthcare system, drawing on theories of collaboration including the influential concept of ‘linkage and exchange’:^16^ Denis and Lomas describe ‘four distinct, but clearly related academic traditions’ that converge on collaborative research^17^ including: action research;^18^ participatory research;^19^ programme evaluation; and knowledge-utilisation research.

Finally, commentators have sought to explain how research engagement at an organisational level can improve the performance of healthcare organisations, drawing on the theoretical approaches that have underpinned efforts to improve organisational performance in healthcare and enhance the design and use of performance management systems. There are well-established literatures on promoting learning organisations, adopting an organisational approach to quality improvement (QI) and knowledge mobilisation.

However while these various literatures provide insights into the review question and pointers to the mechanisms through which research engagement might improve outcomes, the component papers and the reviews based on them do not, by and large, directly consider the benefits of research engagement. Furthermore, until recently research activity was not generally included in the measures used to assess the performance of healthcare organisations, the focus was on measures of activity and cost.^20^

### Scope of the review

Given this wide literature, the scope of the review was carefully considered. Its focus was on studies of practitioner or organisational engagement in research, and the objective was to explore the whole pathway from research engagement to healthcare performance. With this in mind:

- ‘Engagement in research’ was taken to mean a deliberate set of intellectual and practical activities undertaken by healthcare staff (including conducting research and playing an active role in the whole research cycle) and organisations (including playing an active role in research networks, partnerships or collaborations and ensuring the research function is fully integrated into organisational structures). In essence we therefore equated engagement in research with participation in research throughout the research cycle, and this understanding was reflected in the search terms we used in the focused review (see online additional file 1). We noted, however, that the terms ‘engagement in research’ and ‘engagement with research’ are sometimes used interchangeably in the literature. At the start of our review we therefore explored how far a broader definition of research engagement could also include engagement *with* research, taking this term to mean a less substantial involvement at individual and team level related to receiving and transmitting the findings of research. This could include aspects of activities such as continuing medical education (CME), attempts to persuade clinicians to adopt guidelines, and knowledge mobilisation more generally. However, such efforts often focus on encouraging research utilisation alone, and not on research utilisation as an integral phase in the whole research cycle. Given the fact that our brief was to explore the whole pathway from research engagement to healthcare performance, we finally decided to concentrate our resources on the interpretation of research engagement as ‘engagement *in* research’, in the sense given above. We also decided that the scope of our review was already too wide to include the slightly separate although equally important topic of whether or not engaging the public and patients as partners in research improved healthcare performance.
- ‘Healthcare performance’ was understood to reflect the consequences of clinical activity, and was primarily taken to mean improvements in the processes and outcomes of care, rather than other measures of healthcare performance such as efficiency.
- ‘Mechanisms’ were seen in relatively simple terms as levers that instigate and sustain activity, for example, research collaborations between researchers and healthcare staff who are potential users of the findings.

## Methods

**Figure 1:**
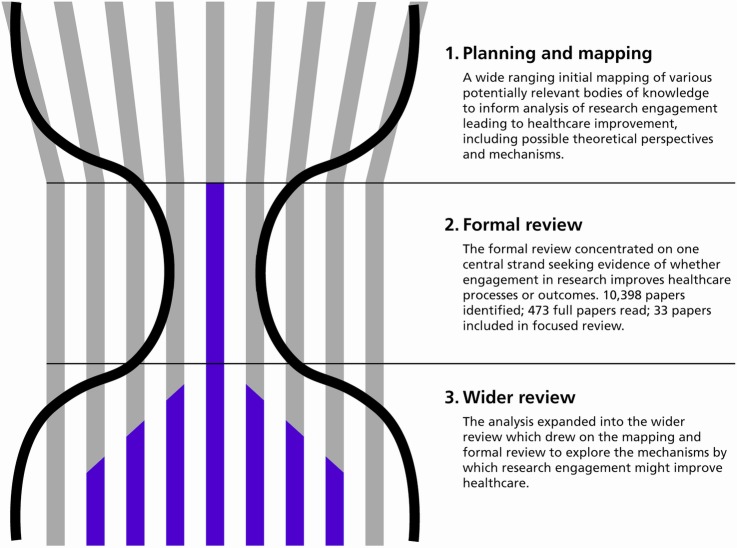
The hourglass shaped three-stage review. Source: Hanney, Boaz, Jones, Soper (2013).^23^

An ‘hourglass’ review was undertaken that consisted of three stages: (1) a broad mapping exercise exploring a large number of bodies of literature that might contain empirical evidence relating to the question and any mechanisms and theoretical perspectives that might be relevant; (2) a focused (or formal) review that concentrated on the core question of whether or not research engagement improves healthcare; and (3) a wider (but less systematic) review of papers identified during the two earlier stages that were relevant to the review question, and included many papers that did not meet the inclusion criteria for the focused review. The hourglass shape refers to the scope of the analysis at each stage, and to the number of papers considered in detail; in terms of the volume of titles and abstracts processed, the throughput of the review was greatest in the second stage (figure 1). A more detailed account of the review approach is available (see online additional file 2) The PRISMA checklist for reporting systematic reviews was followed as far as was feasible.^21^ Ethical approval was obtained from Brunel University’s Research Ethics Committee.

### Stage 1: Planning and mapping

The review team drew on their existing knowledge, initial scans of potentially relevant literatures, team meetings and brainstorming sessions, and on the knowledge and experience of an advisory group of international experts and patient representatives. The latter provided input (largely by email) on the methods used in the review, the literature identified and the findings emerging from the synthesis. This mapping exercise explored the major theoretical approaches that could, potentially, inform the conduct of the review and help to build a framework within which to identify and analyse the mechanisms through which engagement in research can improve healthcare performance. It also informed the choice of the search terms used in Stage 2.

### Stage 2: Focused review

**Figure 2:**
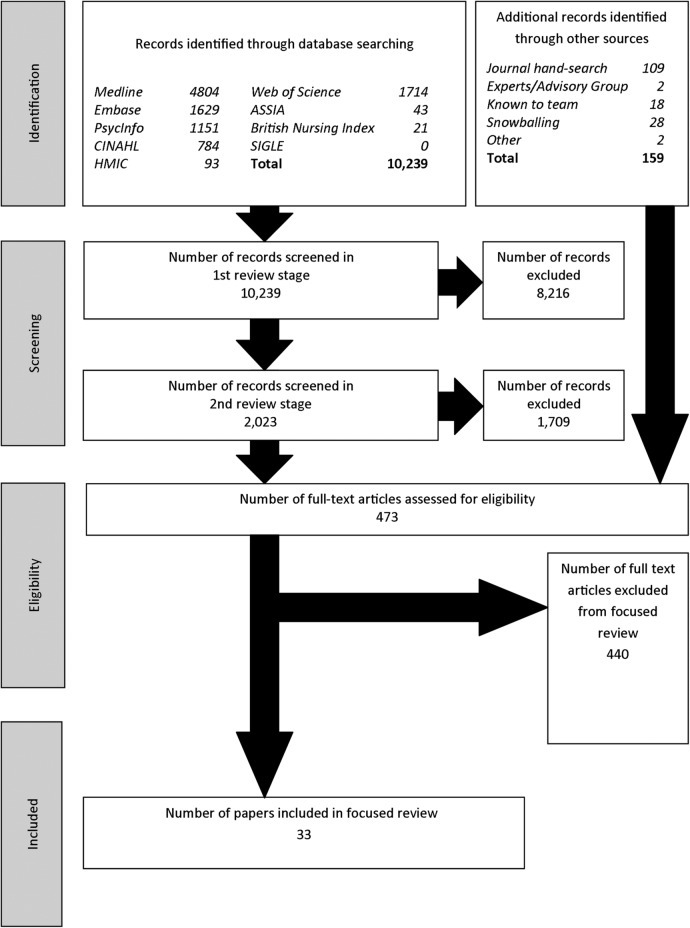
Flow diagram of the literature search for the focused review. Source: Hanney, Boaz, Jones, Soper (2013).^23^

The search strategy involved a comprehensive search of a wide range of relevant databases and sought to identify empirical research studies (not limited to clinical trials)—in which the concept of ‘engagement in research’ was an input and some measure of healthcare ‘performance’ was an output (see figure 2; and see online additional file 1). The search strategy covered the period 1990 to March 2012. MEDLINE, EMBASE, PsycINFO, Cumulative Index to Nursing and Allied Health Literature (CINAHL), Web of Science, Applied Social Sciences Index and Abstracts (ASSIA), British Nursing Index, Health Management Information Consortium (HMIC) and System for Information on Grey Literature in Europe (SIGLE) databases were searched. The database searches were supplemented by manual searching five journals that specialise in this area, by papers suggested by the advisory group, and by snowballing. English language terms were used for searching, although papers identified through this route that were not published in English were considered for inclusion.

The database searches identified 10 239 papers, and 159 were identified from other sources. The searches were conducted by an information scientist, Sarah Jeal advised by Sarah Lawson (a senior information scientist at King's College London), working closely with the review team.

The focused review involved an initial examination of the title of each paper (and the abstract when necessary) to exclude documents that were clearly not relevant. Two or more reviewers then studied the titles and full abstracts in greater depth to assess the eligibility of each paper. Further relevance and quality checks on 473 papers were undertaken to determine whether or not they were suitable to proceed to the data extraction stage. We undertook an initial (broad) test for quality by applying the Dixon-Woods ‘fatally flawed’ test.^22^ This applies the following questions:

- Are the aims and objectives of the research clearly stated?
- Is the research design clearly specified and appropriate for the aims and objectives of the research?
- Do the researchers provide a clear account of the process by which their findings were reproduced?
- Do the researchers display enough data to support their interpretations and conclusions?
- Is the method of analysis appropriate and adequately explicated?

The diversity of methods used in the papers meant that no one quality appraisal tool could be rigidly applied to all papers. The second quality check was, therefore, method specific:

- For RCTs, controlled before and after studies and for qualitative studies we used the relevant checklist provided by the Critical Appraisal Skills Programme (CASP—see: http://www.casp-uk.net/#!checklists/cb36).
- For surveys we used the critical appraisal checklist developed by the Centre for Evidence-based Management (see: http://www.cebma.org/wp-content/uploads/Critical-Appraisal-Questions-for-a-Survey.pdf). This is an approach adapted from a number of existing tools, including: The Pocket Guide to Critical Appraisal (BMJ Books 1996); the BMJ editor's checklists (http://www.bmj.com/about-bmj/resources-authors/article-types/research/editors-checklists) and the checklists of the Evidence for Policy and Practice Information and Co-ordinating Centre (https://eppi.ioe.ac.uk/cms).

The quality of the papers was considered as part of an integrated assessment described as the ‘importance’ dimension discussed below. This was assessed through the quality of the paper, the size of the study and relevance to the focused review question.

#### Analysis

A heterogeneous mix of papers was identified and a standard meta-analysis was not possible. This review involved, therefore, an interpretive rather than an aggregative synthesis.^22^ ^23^ A data extraction sheet was completed for each paper and key aspects of the included studies were collated in a table (table 1). To facilitate analysis, a matrix was developed to characterise the circumstances in which research engagement might improve healthcare performance and the mechanisms that might be at work.

**Table 1:** Findings from the focused review

Author(s) and details of paper	Date	Clinical area or procedure	Country	Level of study	Impact	Finding	Improvement identified
By-product papers							
Adler MW. Changes in local clinical practice following an experiment in medical care: evaluation of evaluation. J Epidemiol Community Health 1978;32:143–6. http://dx.doi.org/10.1136/jech.32.2.143	1978	Inguinal hernia and varicose veins	UK	O	S	+	P
Andersen M, Kragstrup J, Sondergaard J. How conducting a clinical trial affects physicians’ guideline adherence and drug preferences. JAMA 2006;295:2759–64. http://dx.doi.org/10.1001/jama.295.23.2759*	2006	Asthma	Denmark	O	S	-	P
Chen AY, Schrag N, Hao Y, Flanders WD, Kepner J, Stewart A, *et al*. Changes in treatment of advanced laryngeal cancer 1985–2001. Otolaryngol Head Neck Surg 2006;135:831–7. http://dx.doi.org/10.1016/j.otohns.2006.07.012	2006	Cancer (laryngeal)	US	O	B	+	P
Clark WF, Garg AX, Blake PG, Rock GA, Heidenheim AP, Sackett DL. Effect of awareness of a randomized controlled trial on use of experimental therapy. JAMA 2003;290:1351–5. http://dx.doi.org/10.1001/jama.290.10.1351	2003	Apheresis	Canada	C	S?	M -	P
Das D, Ishaq S, Harrison R, Kosuri K, Harper E, Decaestecker J, *et al*. Management of Barrett’s esophagus in the UK: overtreated and underbiopsied but improved by the introduction of a national randomised trial. Am J Gastroenterol 2008;103:1079–89. http://dx.doi.org/10.1111/j.1572-0241.2008.01790.x	2008	Barratt's oesophagus	UK	C	S	+	P
du Bois A, Rochon J, Lamparter C, PFisterer J, and for the Organkommission OVAR. Pattern of care and impact of participation in clinical studies on the outcome in ovarian cancer. Int J Gynecol Cancer 2005;15:183–91*	2005	Ovarian cancer	Germany	O	B	+	HO
Hébert-Croteau N, Brisson J, Latreille J, Blanchette C, Deschenes L. Variations in the treatment of early-stage breast cancer in Quebec between 1988 and 1994. CMAJ 1999;161:951–5	1999	Breast cancer	Canada	O	B	+	P
Janni W, Kiechle M, Sommer H, Rack B, Gauger K, Heinrigs M, *et al*. Study participation improves treatment strategies and individual patient care in participating centers. Anticancer Res 2006;26:3661–7. http://dx.doi.org/10.1016/S0960-9776(05)80107-9	2006	Breast cancer	Germany	O	S	+	P
Jha P, Deboer D, Sykora K, Naylor CD. Characteristics and mortality outcomes of thrombolysis trial participants and nonparticipants: a population-based comparison. J Am Coll Cardiol 1996;27:1335–42. http://dx.doi.org/10.1016/0735-1097(96)00018-6	1996	AMI	Canada	O	S	M+	HO
Jones B, Ratzer E, Clark J, Zeren F, Haun W. Does peer-reviewed publication change the habits of surgeons? Am J Surg 2000;180:566–9. http://dx.doi.org/10.1016/S0002-9610(00)00495-5	2000	Appendectomy	US	C	S	-	P
Karjalainen S, Palva I. Do treatment protocols improve end results? A study of survival of patients with multiple myeloma in Finland. BMJ 1989;299:1069–72. http://dx.doi.org/10.1136/bmj.299.6707.1069	1989	Leucaemia	Finland	O	S	+	HO
Kizer JR, Cannon CP, McCabe CH, Mueller HS, Schweiger MJ, Davis VG, *et al*. Trends in the use of pharmacotherapies for acute myocardial infarction among physicians who design and/or implement randomized trials vs physicians in routine clinical practice: the MILIS-TIMI experience. Multicenter investigation on limitation of infarct size. Am Heart J 1999;137:79–92	1999	AMI	US	C	B	+	P
Majumdar SR, Chang W-C, Armstrong PW. Do the investigative sites that take part in a positive clinical trial translate that evidence into practice? Am J Med 2002;113:140–5. http://dx.doi.org/10.1016/S0002-9343(02)01166-X*	2002	AMI	Canada	O	S	-	P
Majumdar SR, Roe MT, Peterson ED, Chen AY, Gibler WB, Armstrong PW. Better outcomes for patients treated at hospitals that participate in clinical trials. Arch Intern Med 2008;168:657–62. http://dx.doi.org/10.1001/archinternmed.2007.124*	2008	Unstable angina	US	O	B	+	HO
Meineche-Schmidt V, Hvenegaard A, Juhl HH. Participation in a clinical trial influences the future management of patients with gastro-oesophageal reflux disease in general practice. Aliment Pharmacol Ther 2006;24:1117–25. http://dx.doi.org/10.1111/j.1365-2036.2006.03046.x*	2006	Gastro-oesophageal reflux	Denmark	C	S	+	P
Morton AN, Bradshaw CS, Fairley CK. Changes in the diagnosis and management of bacterial vaginosis following clinical research. Sex Health 2006;3:183–5. http://dx.doi.org/10.1071/SH06024	2006	Sexual health	Australia	C	S	M+	P
Pancorbo-Hidalgo PL, Garcia-Fernandez FP, Lopez-Medina IM, Lopez-Ortega J. Pressure ulcer care in Spain: nurses’ knowledge and clinical practice. J Adv Nurs 2007;58:327–38. http://dx.doi.org/10.1111/j.1365-2648.2007.04236.x*	2006	Pressure ulcer	Spain	C	B	+	P
Pons J, Sais C, Illa C, Méndez R, Suñen E, Casas M, *et al*. Is there an association between the quality of hospitals’ research and their quality of care? J Health Serv Res Policy 2010;15:204–9. http://dx.doi.org/10.1258/jhsrp.2010.009125*	2010	AMI	Spain	O	B	+	HO
Rich AL, Tata LJ, Free CM, Stanley RA, Peake MD, Baldwin DR, *et al*. How do patient and hospital features influence outcomes in small-cell lung cancer in England? Br J Cancer 2011;105:746–52. http://dx.doi.org/10.1038/bjc.2011.310*	2011	Small cell lung cancer	UK	O	B	M+	P
Rochon J, du Bois A. Clinical research in epithelial ovarian cancer and patients’ outcome. Ann Oncol 2011;22(Suppl. 7):vii16–19. http://dx.doi.org/10.1093/annonc/mdr421*	2011	Ovarian cancer	Germany	O	B	+	HO
Salbach NM, Guilcher SJ, Jaglal SB, Davis DA. Determinants of research use in clinical decision making among physical therapists providing services post-stroke: a cross-sectional study. Implementation Sci 2010;5:77. http://dx.doi.org/10.1186/1748-5908-5-77	2010	Stroke	Canada	C	B	+	P
Network papers							
** **Abraham AJ, Knudsen HK, Rothrauff TC, Roman PM. The adoption of alcohol pharmacotherapies in the Clinical Trials Network: the influence of research network participation. J Subst Abuse Treat 2010;38:275–83. http://dx.doi.org/10.1016/j.jsat.2010.01.003*	2010	Alcohol-use disorders	US	O	B	+	P
Carpenter WR, Reeder-Hayes K, Bainbridge J, Meyer A-M, Amos KD, Weiner BJ, *et al*. The role of organizational affiliations and research networks in the diffusion of breast cancer treatment innovation. Med Care 2011;49:172–9. http://dx.doi.org/10.1097/MLR.0b013e3182028ff2*	2011	Breast cancer	US	O	B	+	P
Ducharme LJ, Knudsen HK, Roman PM, Johnson JA. Innovation adoption in substance abuse treatment: exposure, trialability, and the Clinical Trials Network. J Subst Abuse Treat 2007;32:321–9. http://dx.doi.org/10.1016/j.jsat.2006.05.021*	2007	Substance abuse	US	O	S	M+	P
Knudsen HK, Abraham AJ, Johnson JA, Roman PM. Buprenorphine adoption in the National Drug Abuse Treatment Clinical Trials Network. J Subst Abuse Treat 2009;37:307–12. http://dx.doi.org/10.1016/j.jsat.2008.12.004*	2009	Substance abuse	US	O	S	+	P
Laliberte L, Fennell ML, Papandonatos G. The relationship of membership in research networks to compliance with treatment guidelines for early-stage breast cancer. Med Care 2005;43:471–9. http://dx.doi.org/10.1097/01.mlr.0000160416.66188.f5*	2005	Breast cancer	US	O	B	+	P
Rhyne R, Sussman AL, Fernald D, Weller N, Daniels E, Williams RL, *et al*. Reports of persistent change in the clinical encounter following research participation: a report from the Primary Care Multiethnic Network (PRIME Net). J Am Board Fam Med 2011;24:496–502. http://dx.doi.org/10.3122/jabfm.2011.05.100295	2011	Acanthosis Nigricans	US	C	S	+	P
Siegel RM, Bien J, Lichtenstein P, Davis J, Khoury JC, Knight JE, *et al*. A safety-net antibiotic prescription for otitis media: the effects of a PBRN study on patients and practitioners. Clin Pediatr 2006;45:518–24. http://dx.doi.org/10.1177/0009922806290567	2006	Otitis media	US	C	S	+	P
Warnecke R, Johnson T, Kaluzny A, Ford L. The community clinical oncology program: its effect on clinical practice. J Qual Improv 1995;21:336–9.	1995	Breast cancer	US	C	S	+	P
Intervention papers							
Chaney EF, Rubenstein LV, Liu C-F, Yano EM, Bolkan C, Lee M, *et al*. Implementing collaborative care for depression treatment in primary care: a cluster randomized evaluation of a quality improvement practice redesign. Implement Sci 2011;6:121. http://dx.doi.org/10.1186/1748-5908-6-121	2011	Depression	US	O	S	M+	P
Goldberg HI, Neighbor WE, Hirsch IB, Cheadle AD, Ramsey SD, Gore E. Evidence-based management: using serial firm trials to improve diabetes care quality. Joint Commission J Qual Improvement 2002;28:155–66	2002	Diabetes	US	O	S	M -	P
Hall C, Sigford B, Sayer N. Practice changes associated with the Department of Veterans Affairs’ Family Care Collaborative. J Gen Int Med 2010;25(Suppl. 1):18–26. http://dx.doi.org/10.1007/s11606-009-1125-3	2010	Rehab for war veterans	US	O	S	+	P
Puoane T, Sanders D, Ashworth A, Chopra M, Strasser S, McCoy D. Improving the hospital management of malnourished children by participatory research. Int J Qual Health Care 2004;16:31–40. http://dx.doi.org/10.1093/intqhc/mzh002	2004	Malnourishment in children	S Africa	O	S	M+	HO

*Paper that made an important contribution to the review (in terms of relevance, quality and size).

−, negative; +, positive; B, broader; C, clinicians; HO, health outcomes; M, mixed; O, organisations; P, processes of care; S, specific.

Adapted from Hanney, Boaz, Jones, Soper (2013)^23^.

This matrix identified two dimensions: the degree of intentionality and the scope of impact. Least intentionality was when the improvement in healthcare performance resulting from engagement in research was a by-product of research that was conducted with the primary aim of testing a specific therapy or approach. Greatest intentionality was when there was an explicit intention to produce improvements in healthcare performance as a direct consequence of research engagement by healthcare staff through interventions such as collaborations, participatory research and/or organisational approaches. Research networks were considered to be in the middle of this spectrum.

Impact is generally defined as a ‘strong effect or influence’,^24^ and it was in this sense that the impact of engagement in research was discussed in the focused review papers—the majority of which sought evidence of a positive association between research engagement and improved healthcare outcomes or processes. Two categories of the scope of impact were identified, broader and specific. Broader impact referred to those who had engaged in research being more willing and/or able to provide evidence-based care that was based on relevant research conducted anywhere, and that was not related to the specific findings of the research in which they were engaged. Specific impact referred to those who had engaged in research being more willing and/or able to provide evidence-based care that was related to the specific findings of the research in which they were engaged.

Each paper that reached the final data extraction step was analysed using this matrix, and also in relation to:

- Importance of the paper to the review. This was based on quality (including, where appropriate, how well controlled the study was), size of the study and relevance to the review question.
- Whether the findings of the paper were positive or negative in relation to the review question (ie, positive if they showed research engagement did improve healthcare, and negative if not). Within each group some were also classified as mixed.
- The level of engagement discussed (clinician or organisational).

### Stage 3: Wider review

The final stage was an informal wider review. This was intended to support the findings of the focused review and explore the mechanisms through which research engagement might improve healthcare, building on relevant theories. All the 440 papers excluded at the full-paper review stage of the focused review were considered for this wider review, plus others identified during the mapping stage and ongoing snowballing exercises. Relevance was determined in relation to the theoretical approaches outlined above and the review team's emerging understanding of the mechanisms involved.

## Results

### Results of the focused review

Table 1 summarises the characteristics of the 33 papers that were included in the focused review. The papers covered 15 clinical fields, including 10 cancer research papers and 6 cardiovascular studies. The papers came from 9 countries, with nearly half (15) from the US and another 5 from Canada.

*Degree of intentionality:* There were 21 by-product papers (least intentionality), 8 network papers (mid-range intentionality) and 4 intervention papers (greatest intentionality). The focused review papers presented in table 1 are organised according to this dimension.

*Importance:* Fourteen papers were identified as important and 19 less important in terms of their contribution to the focused review question. The papers identified as important are starred in table 1.

*Level of engagement:* The ratio of organisational to clinician studies in the by-product and network categories was approximately the same (13v8; 5v3). In contrast, all the intervention studies were at the organisational level. In total, 22 papers were at the organisational level, of which 19 were positive; and 11 at the clinician level, of which 9 were positive.

*Positive papers:* A majority of the papers (28) were positive with regard to whether research engagement improved healthcare performance. However, only a minority of the positive cases (7 out of 28) reported improved health outcomes, the remainder reported improved (usually more evidence-based) processes of care. Among the papers reporting improved health outcomes, two German studies explored the association between hospital trial participation and processes and outcomes such as the use of guideline-indicated care and in-hospital mortality for patients with ovarian cancer. These studies found that overall survival was significantly worse in patients treated in non-study hospitals.^25^ ^26^ Similarly, patients treated for unstable angina in US hospitals participating in clinical trials were found to have significantly lower mortality than those treated in non-participating hospitals,^27^ and a Spanish study of the relationship between bibliometric measures of research output in acute hospitals and hospital mortality for two common cardiac conditions found a low-to-moderate negative correlation between the risk-adjusted mortality ratio and the weighted citations ratio.^28^

Among the papers reporting improvements in the processes of care, a UK study of patients with small-cell lung cancer concluded that patients first seen at a hospital with a keen interest in clinical trials are more likely to receive chemotherapy,^29^ and two US studies of patients treated for breast cancer at facilities that were members of cancer research networks found that they were more likely to receive guideline-concordant treatment or be given innovative treatment offering promise.^30^ ^31^ This latter finding about the positive influence of involvement in research networks on organisational innovation was also confirmed in three US studies on alcohol and substance abuse.^32–34^

*Impact:* Taken together, the papers were divided almost equally into those with a broader impact on healthcare performance (16) and those with a more specific impact (17) (although all the intervention studies described a specific impact). Within this overall balance, 13 of the 28 positive studies described a broader impact and these included 10 out of the 17 positive by-product studies.

### Results of the wider review

More than 80 papers included in the wider review reported on studies that illustrated some progress along the pathway from research engagement to improved healthcare (but that had not gone far enough to be included in the focused review). The wider review provided further evidence to support the findings of the focused review about the nature of the relationship between research engagement and healthcare outcomes, the mechanisms involved and the role of the context provided by health organisations and systems.

## Discussion

Overall on the basis of the analysis of the papers in the focused review, it is reasonable to suggest that when clinicians and healthcare organisations engage in research there is the likelihood of improvement in their healthcare performance, even when that has not been the primary aim of the research. This evidence related mainly, though not exclusively, to improvement in the processes of care rather than in health outcomes.

### What these findings indicate about the mechanisms involved

The 21 ‘by-product’ papers with the degree of least intentionality constituted the largest group in the focused review (see table 2). In these papers the main purpose of the original research engagement (by clinicians or organisations) was to conduct or participate in research studies to evaluate new therapies, procedures, etc. The by-product papers were separate studies, usually conducted later, that explored the impact on healthcare that had arisen as ‘by-products’ of the research engagement in the original study and sometimes analysed, or speculated about, what might have caused this impact. In the sense used in this review, the term ‘by product’ is therefore in some circumstances associated with the concept of ‘absorptive capacity’. There were 17 positive ‘by-product’ papers, 10 reporting a broad impact (the use of research findings from wherever they come) and 7 reporting a more specific impact (use of research findings from a specific study in which the research engagement occurs). The discussions in these papers suggest that at clinician and organisational levels different mechanisms—such as changes in clinicians’ attitudes and behaviour or the long-term use of infrastructure created to support a particular trial—may be at play (see table 2).

**Table 2:** Mechanisms through which research engagement may improve healthcare performance

	Mechanisms identified in the focused review		Insights from the wider review
By product papers			
	Broad impact	Specific impact	Research-active staff may differ from their peers in non-research-active settings because of: personal characteristics, multidisciplinary collaboration, additional training and education or specialisation An increasing recognition of the ‘by-product’ type benefits from research engagement has encouraged further thinking about how best to build on and regularise these opportunities
Clinician	Change in attitudes and behaviour that research engagement can promote Involvement in the processes of research	▸ Greater awareness and understanding of the specific research findings	
Organisation	▸ Use of the infrastructure created to support trials more widely, or for a longer period, to improve patient care	▸ Applying the processes and protocols developed in a specific study (not counting any impact from regimens in the intervention arm) to all patients with specific illness, irrespective of their involvement in the trial	
Network papers			
Clinician		Increased relevance of the research Increased knowledge and understanding of the findings gained through participation in the research Clinician participation in research networks particularly effective when the science is changing rapidly and when keeping up-to-date is critical	Mechanisms such as practice facilitators, project development meetings and network convocations allow two-way knowledge exchange throughout a research network, enabling clinicians to engage with question generation and the resulting research, and ensuring that the research is more relevant to practitioners Limitations about what can be achieved by research networks Need for a supportive context that enables clinicians and their organisations to participate in research and research networks Evidence of a growing international interest in the benefits that might come from research networks
Organsational	Centres within networks build up a record of implementing research findings Network membership increases the likelihood of physicians recommending guideline concordant treatment Organisations affiliated to a network adopt an integrated, programmatic approach to improving the quality of care, including the professional education, training and national meetings provided		
Intervention papers			
Organisation		▸ The importance of effective collaboration and the need for a supportive context	Healthcare organisations and systems provide the context within which research engagement operates at other levels Organisations in which the research function is fully integrated into the organisational structure can out-perform other organisations that pay less heed to research and its outputs

The second largest group of papers in the focused review was the eight network papers, which described the situation broadly in the middle of the spectrum of intentionality. All these papers came from the US, reflecting the more established nature of formal research networks in the USA, and also an approach to evaluation that is consistent with the inclusion criteria used for the focused review. All the network papers were positive, and the mechanisms discussed by the authors represent a partial formalisation and use on a regular basis (through the provision of more effective collaboration and more supportive contexts) of those mechanisms discussed in the by-product papers (see table 2).

The partial formalisation and the importance of context identified in the network papers were taken still further in interventions deliberately designed to integrate the research function into organisational structures. These were described in the four intervention papers in the focused review,^35–38^ and included collaborative approaches, quality improvement research initiatives, participatory and action research, and organisational approaches where the intention was explicitly to produce improvements in healthcare performance as a direct consequence of the research engagement of the organisation. One of these studies was positive,^37^ two were mixed/positive^35^ ^38^ and one was categorised as mixed/negative^36^ because the improvements that were achieved during the intervention project were later reversed. Most of the improvements described were in healthcare processes, although improvements in health outcomes were reported in one study.^38^ These four intervention papers largely described the adoption of the specific research that featured in the intervention. However, they also raised issues about how broader impact can be achieved throughout an organisation which resonate with how research networks operate, such as the importance of effective collaboration (see table 2).

### The formalisation of engagement by clinicians and healthcare organisations in research

The largest group of the papers categorised as important in the focused review were those in which the level of engagement considered was organisational and the scope of impact discussed was broad. This finding ties in with the increasing formalisation of attempts to promote what were hitherto often viewed as the ‘by-product’ benefits of research engagement. This formalisation is exemplified in recent initiatives designed to promote clinical research and to encourage the translation of research such as the development of research networks, the NIH Road Map in the USA,^11^ the NIHR Collaborations for Leadership in Applied Health Research and Care in the UK.^12^ In an important realignment of objectives, these moves towards trials and other well-found research taking place within networks and as part of wider interventions mean that increasingly research engagement leading to improved healthcare performance is shifting from being a by-product to an intended outcome of research funding. To date the effect of institutional research activity on patient outcomes and, specifically, the organisational factors that can facilitate or hinder provider participation in research and that underpin implementation effectiveness have not been investigated extensively.^39^ ^40^ However, there was evidence from the wider review that initiatives such as those described above are beginning to result in progress being made along the pathway from research engagement to improved healthcare.^41^ And this evidence has since been supplemented by more recently published papers that report that research active UK NHS Trusts have lower risk-adjusted mortality for acute admissions^42^ ^43^ (a conclusion that supports the Spanish-based findings of the earlier focused-review paper by Pons *et al*^28^), and that describe the positive outcomes being achieved by initiatives such as the UK NIHR CLAHRCs^44^ ^45^ and the US NIH's Clinical and Translational Science Awards.^46^

### The nature of the relationship between research engagement and healthcare improvement

Throughout this review the term ‘impact’ was used to mean the influence or effect that research engagement might have on healthcare improvement. The nature of this relationship was discussed in some (though not all) of the papers in the focused review whose authors identified various measures of research activity (such as recruitment to trials^27^ or production of peer-reviewed papers^28^ or affiliation to a research network^30^ ^32^), highlighted confounding factors (such as hospital teaching status^28^), and undertook multivariate analysis to establish the nature and strength of any association.^30^ ^32^

Overall, it is clear that at both clinician and organisational levels engagement in research differs in intensity and in form, operates through a variety of mechanisms, and is only one of many influences on performance. Although for the reasons given above, the literature on ‘engagement *with* research’ did not fall within the inclusion criteria for this review, this literature is considerably more established than the literature on ‘engagement *in* research’, and has interesting parallels with the conclusions of our own review. In particular, both literatures recognise that there is no single magic bullet^47^ and that there is a need for multiple parallel strategies to encourage engagement both with and in research in order to improve healthcare performance.^48^

Evaluating the effect of *active* engagement in research of the sort identified in this review is, therefore, not ‘a trivial issue’.^26^ For example, at organisational level one measure of research engagement is the extent of patient enrolment in trials. However, healthcare organisations that participate actively in trials may have other institutional characteristics that also improve patient outcomes, such as a high volume of patients, well-respected training programmes and well-motivated, highly educated staff; and there is considerable potential for confounding.^27^ In order to establish an association between research engagement and improvement in healthcare, it is, therefore, necessary for studies to adjust for such institutional characteristics,^4^ and for other attributes such as organisational culture.^41^ Having established an association, further data on whether this effect increases with higher levels of participation^27^ and over the time an institution is research-active are needed to provide evidence of causation.

Disaggregating how the various mechanisms through which research engagement improves performance operate in complex healthcare systems and factoring the role of ‘organisational form’ into all this^41^ is also not straightforward.^40^ ^49–51^ Both the focused and the wider reviews identified situations in which impacts seemed less likely to arise from research engagement, and in which the operation of networks and schemes aimed at involving clinicians more fully in research faced difficulties in making progress, particularly when there were not changes at the organisational level to support these initiatives.^36^ ^52^ This suggests that, if we are to understand better why “…healthcare institutions or service providers who are active in research deliver better care and outcomes than those who do not participate in clinical research?”,^4^ more work is needed to encourage engagement both in and with research in order to identify the organisational determinants of implementation effectiveness and thereby improve healthcare performance. This might, for example, mean building on the work undertaken by Teal *et al*^41^ in which they used an organisational model of innovation implementation that identified six factors that facilitate or hinder implementation: an organisation's readiness for change, the level of management support and resources available, the implementation policies and practices that the organisation puts into place, the climate for implementation that results from these policies and practices, and the extent to which intended users of the innovation perceive that innovation use fosters the fulfilment of their values. Or exploring further the insights developed by the US Veterans Health Administration that suggest that having researchers nested in a fully integrated healthcare delivery system with a stable patient population that has an exceptionally high prevalence of chronic conditions provides them ‘with unparalleled opportunities to translate research questions into studies and research findings into clinical action’.^53^ A different but equally promising approach is the use of a form of statistical analysis—mediation analysis—to assess the mediating effect of various clinical pathways on the impact of research activity on patient outcomes.^40^

#### Limitations

Many bodies of literature address the broad question of whether research engagement improves performance, but most published papers do so tangentially. The initial mapping stage therefore sought to identify papers published in different fields, journals and countries, and a significant amount of time had to be dedicated to this and to refining the question and developing search terms. The focused review shared the limitations of other systematic reviews in that it inevitably excluded large volumes of potentially interesting, relevant research that did not meet the inclusion criteria or that provided too little information about key elements of the study (such as design and outcomes). In particular, studies assessing the impact made on clinician behaviour by small, locally conducted pieces of research were difficult to interpret without full knowledge of the context. A wider additional synthesis (the wider review) was undertaken to support the findings of the focused review and give the final review more explanatory power. Another common limitation in systematic reviews is the reliance of reviewers on what is already published in the literature, and one result of this was that the section of the focused review on networks drew exclusively on US studies of research networks. Linked to this is another challenge common to systematic reviews: the impact of publication bias and, specifically, towards the publication of studies with positive results. This was addressed by searching the grey literature, conducting a web search and writing to some key authors in the field to identify unpublished literature.

## Conclusion

Systematic analysis of the data related to the engagement by clinicians and healthcare organisations in research is in its infancy, despite widely held assumptions about the benefits of this engagement. The focused review reported above concluded that there *is* some positive evidence (albeit limited) that engagement by clinicians and healthcare organisations in research can improve healthcare performance. However, although the focused review also identified a range of mechanisms through which engagement by clinicians and healthcare organisations in research might result in improved healthcare performance, and the wider review added additional evidence, it remains unclear how these effects are produced.

Overall what was clear, however, is that there are many circumstances and mechanisms at work, more than one mechanism is often operative and the evidence available for each one is limited. These mechanisms overlap and rarely act in isolation, and their effectiveness depends on the context in which they operate. The number of research networks is growing, and the contribution of collaborative approaches to research is also developing. At an organisational level there is an increasing formalisation of potential mechanisms, and research processes themselves have become an important means through which research engagement can improve healthcare performance. Allied to these developments there is a need for further empirical research, including more fine-grained organisational studies that consider not only the research engagement of all the relevant actors but also the organisational determinants of implementation effectiveness.
